# Intertumoral Heterogeneity in Multifocal Breast Cancer Mimicking a Collision Tumor on Imaging: A Case Report

**DOI:** 10.7759/cureus.94235

**Published:** 2025-10-09

**Authors:** Yoshika Nagata, Izumi Kinoshita, Toshihiro Saeki, Daiji Uchiyama, Takahisa Fujikawa

**Affiliations:** 1 Surgery, Kokura Memorial Hospital, Kitakyushu, JPN; 2 Pathology, Kokura Memorial Hospital, Kitakyushu, JPN

**Keywords:** breast cancer, heterogeneity, intrinsic subtype, multifocal breast cancer, surgical resection

## Abstract

Breast cancer exhibits heterogeneity characterized by intertumor heterogeneity, where distinct lesions present different subtypes, and intratumor heterogeneity, where a single tumor evolves over time. We present a unique case of synchronous, adjacent multifocal breast cancers demonstrating histological findings of distinct molecular subtypes. A 72-year-old woman with a history of ovarian cancer and a family history of breast cancer presented with a right breast lump. Imaging showed two contiguous but distinct lesions. Core needle biopsy identified invasive ductal carcinoma. Immunohistochemistry revealed luminal B and luminal B/human epidermal growth factor receptor 2 (HER2)-positive subtypes. Total mastectomy and sentinel lymph node biopsy were performed, and pathology confirmed two partially fused tumors separated by fibrous stroma. The HER2-positive component demonstrated higher proliferative activity and nuclear grade. Postoperatively, the patient received chemotherapy, anti-HER2 therapy, and remains disease-free on endocrine therapy. This case highlights a rare breast cancer presentation with identical histology but different molecular subtypes mimicking a collision tumor on imaging. It underscores the clinical relevance of tumor heterogeneity and the importance of combining imaging, pathology, and molecular profiling for accurate diagnosis and tailored treatment.

## Introduction

Breast cancer shows heterogeneity: intertumor heterogeneity when separate lesions have different subtypes, and intratumor heterogeneity when a single tumor evolves over time [1,2]. A collision tumor is the combination of two histologically distinct neoplasms in a single location [3]. The occurrence of collision tumors in the breast is very rare, with cases reported for a combination of “breast cancer and leukemia” and “breast cancer and phyllodes tumor” [4,5].

Here, we report the case of a woman diagnosed with multifocal synchronous breast cancers that exhibited a nearly identical histological classification, yet different subtypes. This report emphasizes the importance of imaging and histopathological evaluation in diagnosing and treating such cases.

## Case presentation

A 72-year-old woman visited our hospital complaining of a right breast lump. She had a medical history of ovarian serouscarcinoma, hypertension, and gallbladder polyps. Her family history was notable for breast cancer in her sister. Physical examination revealed an elastic, hard lump with irregular margins located in the upper-inner quadrants of the right breast. No skin changes or dimpling were observed. There were no inflammatory signs or palpable lymphadenopathy. A red blood cell count of 3.9 million/µL (reference range: 3.8-5.4 million/µL), a hemoglobin level of 11.2 g/dL (reference range: 11.5-15.9 g/dL), and a hematocrit (Ht) of 34.4% (reference range: 34.0-45.0 %) were found in the blood, which all pointed to mild anemia. Both tumor markers were within normal limits, with a carcinoembryonic antigen level of 1.8 ng/mL (reference range: <5.0 ng/mL) and a cancer antigen 15-3 level of 5.2 U/mL (reference range: <31.3 U/mL). These data were shown in Table 1.

**Table 1:** Laboratory findings. RBC: red blood cell; Hb: hemoglobin; Ht: hematocrit; CEA: carcinoembryonic antigen; CA15-3: cancer antigen 15-3

Parameter	Result	Reference range
RBC	3.9 × 10^6^/µL	3.8–5.4 × 10^6^/µL
Hb	11.2 g/dL	11.5–15.9 g/dL
Ht	34.4%	34.0–45.0%
CEA	1.8 ng/mL	<5.0 ng/mL
CA15-3	5.2 U/mL	<31.3 U/mL

**Figure 1:**
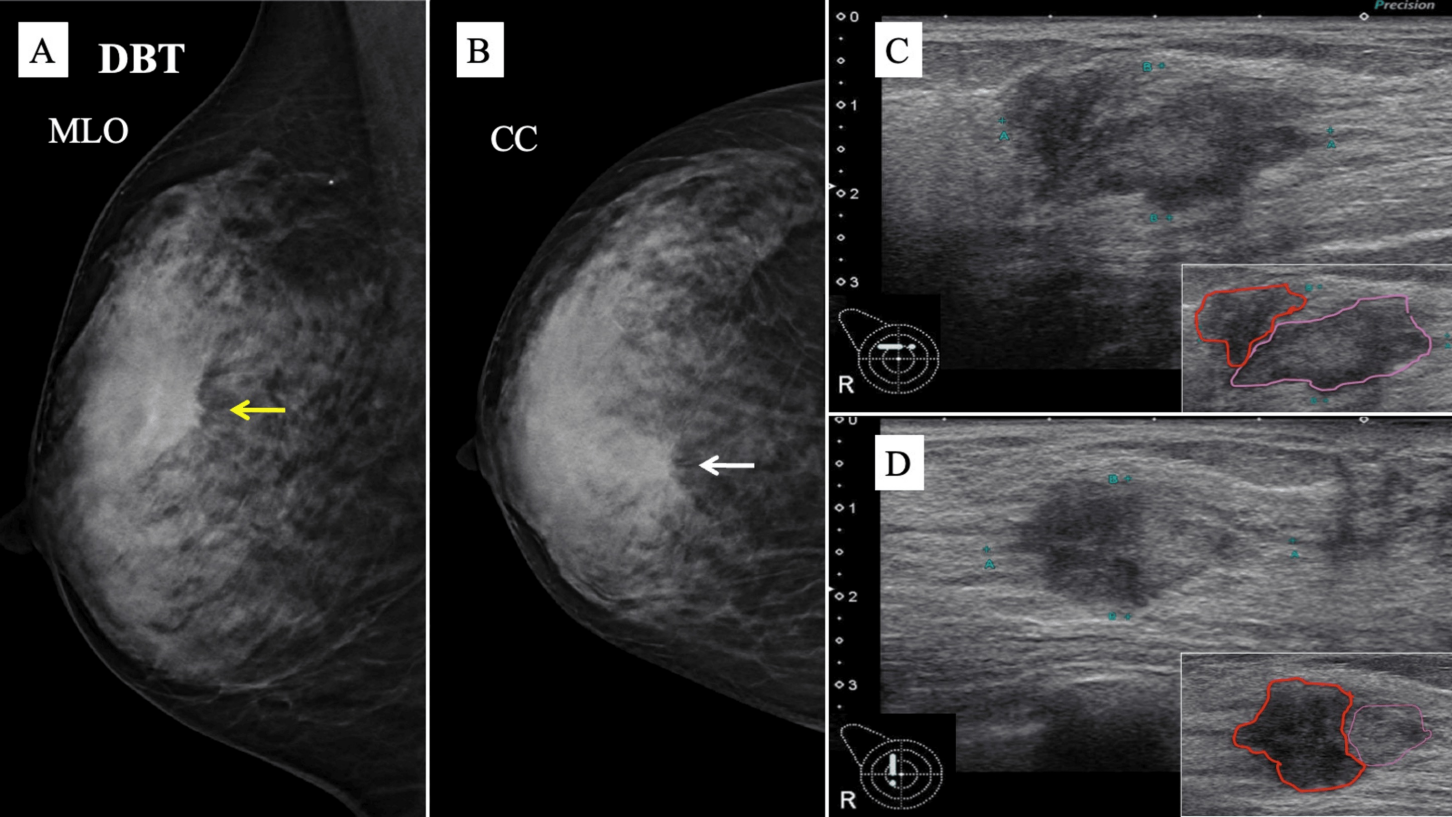
MG and US findings. (A) DBT shows a high-density spiculated mass (yellow arrow) on the MLO view. (B) A CC view of DBT reveals architectural distortion surrounding the mass (white arrow). (C, D) US findings. The transverse US image in C reveals two adjacent irregular hypoechoic masses. The internal echoes were slightly different in the longitudinal view, as shown in D. The tumor margins are highlighted in pink and red in the lower right insets of C and D, respectively. The pink-outlined tumor is HER2-negative, and the red-outlined tumor is HER2-positive breast cancer. Tumors outlined in red have lower internal echo values. CC: craniocaudal; DBT: digital breast tomosynthesis; HER2: human epidermal growth factor receptor 2; MG: mammography; MLO: mediolateral oblique; US: ultrasonography

Digital breast tomosynthesis, also known as three-dimensional mammography (MG), showed a high-density spiculated mass with distortion in the middle-inner region of the right breast (Figures 1A, 1B). The breast was dense, with some unclear boundaries, making it appear as a single tumor. No associated calcifications were found within the tumor. Ultrasonography (US) revealed two irregular hypoechoic masses in a continuous configuration. The internal echo density of each mass was variable, and the borders showed a mixture of well-defined and indistinct regions (Figures 1C, 1D). The combined size of the two tumors was 3.1 × 3.0 × 1.7 cm in diameter, and ductal dilatation was observed from the tumor to the nipple.

**Figure 2:**
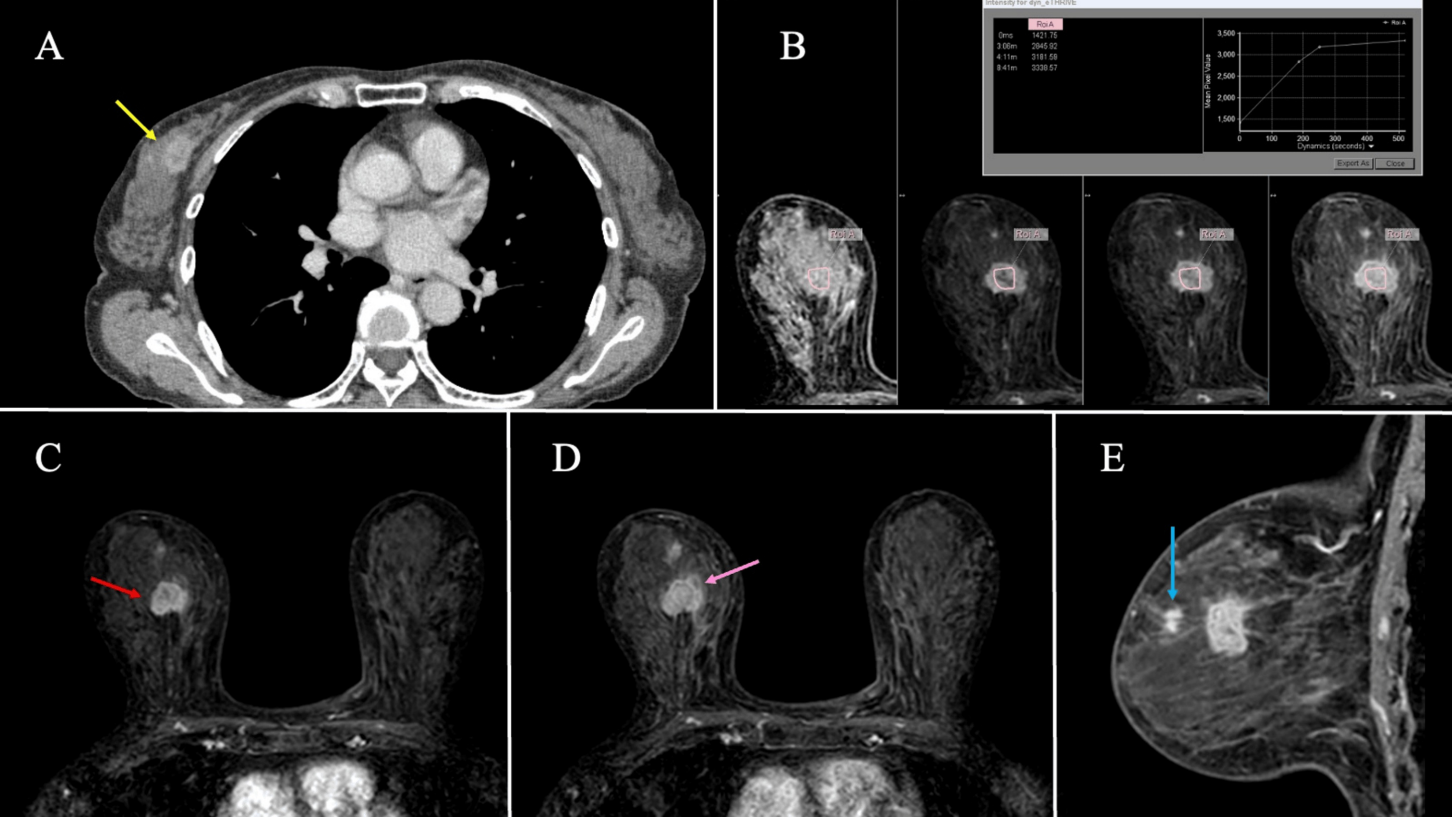
Contrast-enhanced CT and MRI findings. (A) Contrast-enhanced CT demonstrating a dumbbell-shaped mass with variable contrast enhancement in each component (yellow arrow). (B-D) MRI in the axial view: (B) time-intensity curve demonstrating a fast-to-plateau pattern; (C) early strong enhancement in the lateral portion of the mass (red arrow); (D) progressive delayed enhancement in the medial portion (pink arrow). (E) MRI in the sagittal view also depicts a focal lesion toward the nipple, suggestive of a possible daughter lesion (blue arrow).

A contrast-enhanced CT showed a dumbbell-shaped enhancing mass with different levels of contrast within each tumor, leading to speculation that two tumors with different characteristics were colliding (Figure 2A). There were no visible signs of systemic metastases or axillary lymph node enlargement. On MRI, the tumor demonstrated low signal intensity on T1-weighted and T2-weighted fat-suppressed images, with a decreased apparent diffusion coefficient. Contrast-enhanced MRI revealed a high-intensity mass in the right breast. A focal lesion was observed in the direction of the nipple, which could not be excluded as a daughter lesion. The tumor showed heterogeneous enhancement: the lateral portion demonstrated strong early enhancement, while the medial portion showed progressive delayed enhancement. Overall, the time-intensity curve showed a fast-to-plateau pattern (Figures 2B-2E).

Core needle biopsy (CNB) showed an atypical epithelium with a schirrhous pattern, proliferating in cord-like and nodular patterns, and revealed invasive ductal carcinoma of no special type. Immunohistochemical (IHC) analysis revealed that the tumor was heterogeneous and consisted of two distinct components. One component exhibited high estrogen receptor (ER) expression (95%) but lacked progesterone receptor (PgR) expression (5%), with a human epidermal growth factor receptor 2 (HER2) score of 1+ and a Ki-67 index of 20%. The second component demonstrated high ER expression (95%) with moderate PgR expression (15%), HER2 overexpression (score 3+), and a Ki-67 index of 20%. Based on these results, the tumors were diagnosed as luminal (Lum) B and Lum B/HER2-positive subtypes of breast cancers. These IHC data are shown in Table 2.

**Table 2:** Immunostaining results for needle biopsy specimens. Lum: luminal; ER: estrogen receptor; PgR: progesterone receptor; HER2: human epidermal growth factor receptor 2; Ki-67: proliferation marker

Component	ER (%)	PgR (%)	HER2 (score)	Ki-67 (%)
Lum B	95%	5%	1+	20%
Lum B/HER2-positive	95%	15%	3+	20%

**Figure 3:**
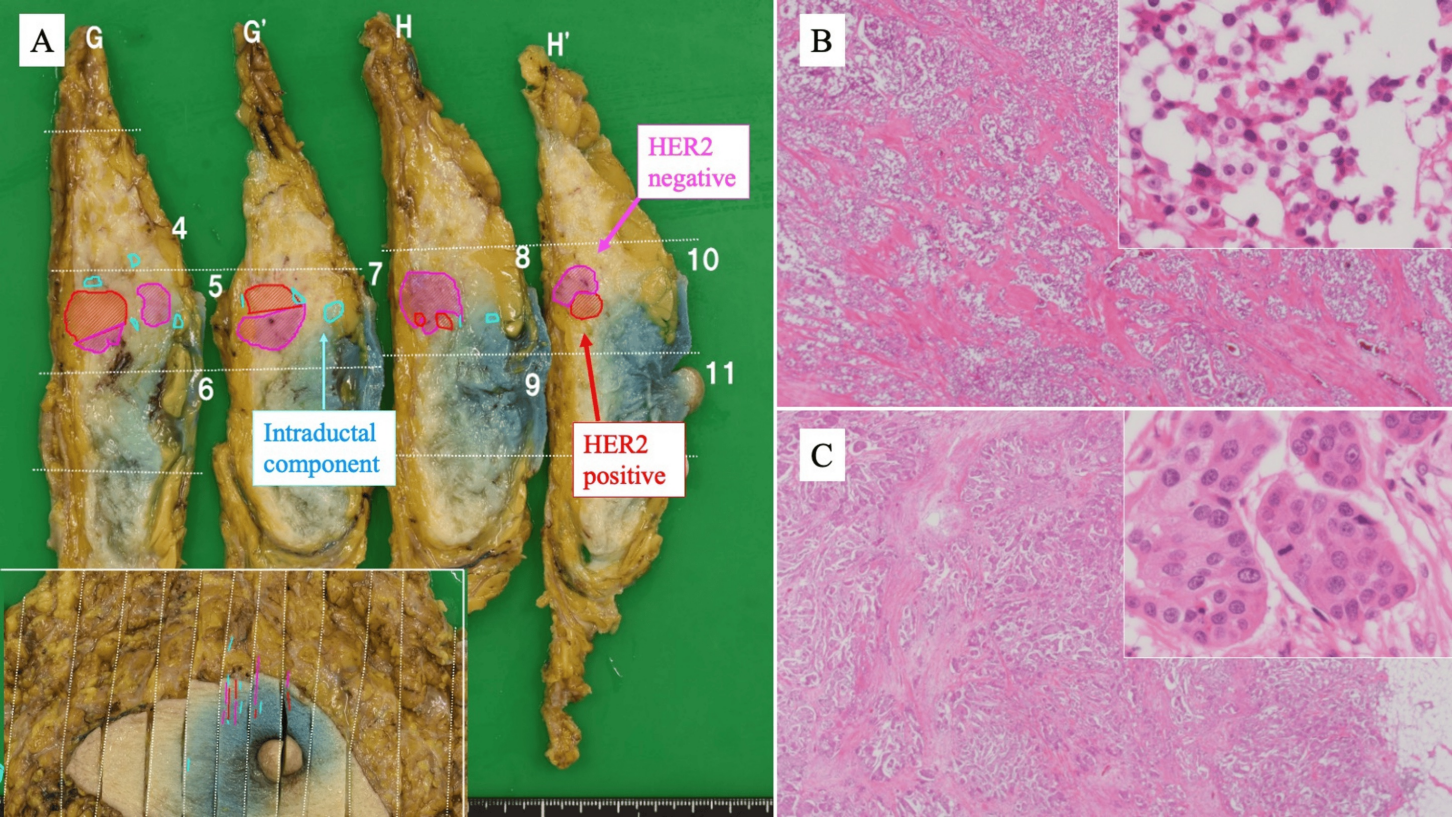
Surgical specimen and HE staining. (A) Gross specimen showing invasive ductal carcinoma with both HER2-negative and HER2-positive components arranged in a spiral pattern, as illustrated by mapping. Intraductal extension toward the nipple is identified, and the spatial relationship between the nipple and the tumor is shown in the inset (lower left). (B) Low-power HE of the HER2-negative component; inset (upper right) shows higher magnification. (C) Low-power HE of the HER2-positive component; inset (upper right) shows higher magnification. Tumor cells in the HER2-negative area appear smaller than those in the HER2-positive area. HE: hematoxylin and eosin; HER2: human epidermal growth factor receptor 2

The clinical stage, according to the Union for International Cancer Control (UICC) TNM classification (8th edition), was cT2N0M0, stage IIA. Although IHC staining revealed diversity among the subtypes, no clear morphological differences were observed in the collected tissues. The possibility of hereditary breast and ovarian cancer (HBOC) syndrome caused by a *BRCA* gene mutation was suggested, but genetic testing was not possible due to her age and budget. Surgical resection (total mastectomy and sentinel lymph node biopsy) was performed based on the family history and patient preference. Macroscopic examination of the surgical specimen revealed a yellowish-white irregular mass with an indistinct border between the two adjacent masses. The specimen revealed invasive ductal carcinoma with a spiral growth pattern (Figure 3A). Hematoxylin and eosin (HE) staining revealed a clear demarcation between the two tumor subtypes, separated by fibrous stroma (Figures 3B, 3C).

**Figure 4:**
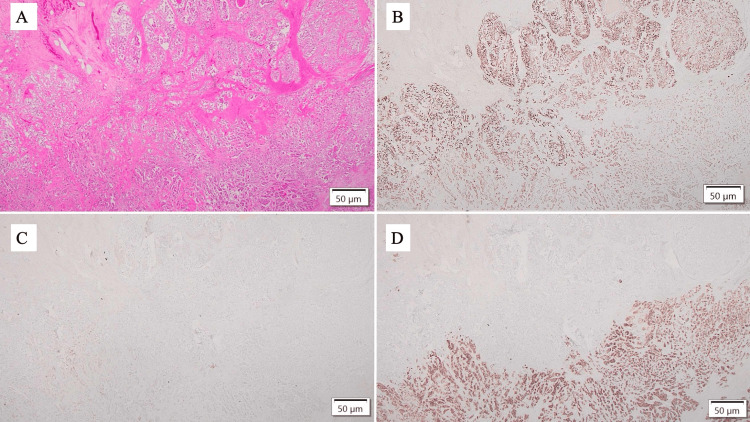
Immunohistochemical staining of the tumor (scale bar: 50 μm). (A) HE. (B) ER showing nuclear positivity. (C) PgR showing low positivity. (D) HER2 showing strong membranous positivity. The lower right corresponds to the Lum B/HER2-positive component, whereas the upper left represents the Lum B component. ER: estrogen receptor; HE: hematoxylin and eosin; HER2: human epidermal growth factor receptor 2; Lum: luminal; PgR: progesterone receptor

IHC analysis confirmed the subtypes as Lum B and Lum B/HER2-positive, consistent with the findings from the CNB (Figures 4A-4D).

Following HER2 IHC confirmation, cellular morphology was re-evaluated. The Lum B component showed a nuclear grade (NG) of 2, composed of a nuclear atypia (NA) score of 2 and a mitotic count (MC) score of 2 (nine mitoses per 10 high-power fields (HPFs)). In contrast, the Lum B/HER2-positive component exhibited higher proliferative activity, with 23 mitoses per 10 HPFs, corresponding to an NG of 3 (NA 2 + MC 3). The tumor cells in the Lum B component appeared smaller than those in the Lum B/HER2-positive component (Figures 3B, 3C). Therefore, it was thought that two breast cancers with different characteristics had coincidentally developed and grown in the same location and had a morphology similar to that of a collision tumor. The final pathological diagnosis, according to the UICC TNM classification (8th edition), was pT2N0M0, stage IIA. The patient received four cycles of epirubicin plus cyclophosphamide followed by four cycles of docetaxel in combination with trastuzumab as postoperative adjuvant therapy. Trastuzumab was then continued as monotherapy every three weeks. Subsequently, endocrine therapy with an aromatase inhibitor was initiated. At the most recent follow-up, four years after surgery, the patient remains disease-free.

## Discussion

Tumor heterogeneity describes the variation in genetic, molecular, and phenotypic features. This can occur within a single tumor (intratumoral heterogeneity) or between different tumors in the same patient or across different patients (intertumoral heterogeneity) [1,2,6]. Temporal intratumoral heterogeneity results in alterations of molecular subtypes between primary and recurrent breast cancers, even when originating from the same histological background. Tumor evolution, therapeutic intervention, or heterogeneity of the tumor microenvironment can alter ER, PgR, or HER2 status [7,8]. It has significant implications for cancer diagnosis, prognosis, and treatment, and contributes to drug resistance and tumor progression.

Collision tumors are characterized by the coexistence of two different histological types within a single lesion, as reported by Spagnolo et al. [9]. In the breast, they are rarely reported and typically involve carcinoma with another histologic type [4,5]. Several hypotheses have been described for the origin of collision tumors, including coincidence, shared tumorigenic environment, tumor evolution, and therapy-induced tumors. With the progression of tumor heterogeneity, some clones adopt different tumor morphologies and form collision tumors. Our case, by contrast, presented with two lesions of identical histology (both invasive ductal carcinoma of no special type), but with different molecular subtypes. Such cases are more commonly classified as synchronous multiple breast cancers.

Breast cancer subtypes have unique imaging characteristics. US studies show that triple-negative (TN) breast cancers tend to have compressive margins, while Lum subtypes have invasive margins. In addition, HER2-positive cancers often have a ductal pattern. Findings such as peritumoral halo are more common in Lum subtypes, while punctate echogenic foci and cystic structures show significant associations with HER2-positive and TN cancers, respectively. On MG images, both Lum B/HER2-positive and HER2-positive subtypes had a higher incidence of calcification compared to other subtypes [10]. On contrast-enhanced MRI, Lum A cancers typically show irregular margins with spiculated edges, while Lum B cancers lack a distinctive imaging pattern. HER2-positive cancers exhibit extensive lesions, while TN cancers appear as round lesions with homogeneous internal features. Breast MRI is more effective in detecting multifocal and/or multicentric disease as well as lymph node involvement in Lum B and HER2-positive subtype breast cancers [11]. In this case of MG, there was no calcification within the irregular tumor mass, but MRI showed multiple densely stained tumor masses adjacent to each other, which was consistent with the imaging findings of a mixture of Lum B and HER2 positivity.

Molecular subtype discordance is not uncommon, occurring in 10% to 25% of cases. A report analyzing molecular subgroups of tumor pairs with bilateral disease found greater subtype discordance in metachronous TN cases separated by a 10-year interval [12]. In addition, discordance rates of ER, PgR, and HER2 expression status have been reported between primary and recurrent breast cancer lesions [13], and patients who convert to ER-negative status have a poor prognosis. Therefore, the European Society for Medical Oncology Clinical Practice Guidelines emphasize the importance of reevaluating hormone receptor and HER2 expression in metastatic lesions.

Importantly, recent studies of MF/MC breast cancer have demonstrated that intertumoral heterogeneity among foci is not uncommon, with discrepancies in pathological type, histological grade, and molecular subtype reported in up to 8-10% of cases. Tong et al. emphasized that evaluating only the largest lesion may underestimate tumor burden and overlook biologically relevant heterogeneity, which could affect staging, treatment selection, and outcomes [14]. Thus, a comprehensive assessment of each lesion, including IHC and molecular characterization, is crucial for accurate management in MF/MC breast cancers.

Another consideration in this case is the patient’s prior history of ovarian cancer. She underwent oophorectomy and treatment at age 54, before menopause, which may have altered her hormonal balance and contributed to subsequent differences in breast cancer subtypes. Moreover, HBOC increases the likelihood of multifocal and synchronous breast cancers; in a cohort of *BRCA* carriers, MF/MC disease occurred in 25% overall and was more than twice as frequent in *BRCA2* than in *BRCA1* [15]. She did not consent to genetic testing due to financial constraints. The potential impact of such hormonal and genetic factors on HER2 expression remains speculative.

Given that treatment strategy and prognosis can be influenced by intertumoral heterogeneity, each lesion should be evaluated individually and reported accordingly. In clinical practice, when multiple breast lesions with differing imaging or morphological features are present, it is essential to biopsy each lesion separately. Sampling only one focus may miss clinically significant components, such as HER2-positive disease, which could critically alter systemic treatment strategies. In our case, identification of HER2 positivity in one lesion directly guided the administration of anti-HER2 therapy. Therefore, clinical management should depend on accurately characterizing intertumoral heterogeneity. Treatment decisions should be based on the most aggressive molecular subtype identified and supported by a multidisciplinary integration of imaging, pathology, and molecular findings.

## Conclusions

This case presents multiple synchronous breast cancers of different subtypes resembling collision tumors on imaging, highlighting the complexity of differentiating intratumoral heterogeneity. The patient underwent comprehensive postoperative adjuvant therapy for each Lum and HER2 subtype and progressed well without recurrence. A multidisciplinary approach combining imaging, pathology, and molecular analysis is essential to advance diagnostic and therapeutic strategies in the management of complex cases exhibiting tumor heterogeneity.
